# Collaborating to Improve Outcomes in Congenital Heart Disease: The Pediatric Heart Network Experience

**DOI:** 10.32604/chd.2025.073995

**Published:** 2026-02-10

**Authors:** Bryanna N. Schwartz, Victoria L. Pemberton, D’Andrea Freemon, Kristin M. Burns, Gail D. Pearson

**Affiliations:** Division of Cardiovascular Sciences, National Heart, Lung, and Blood Institute, National Institutes of Health, Bethesda, MD 20892, USA

**Keywords:** Clinical trial, congenital heart disease research, nursing research, registries

## Abstract

**Background:**

In the 1990s, there were few multicenter research collaborations and pediatric cardiovascular clinical trials. The National Heart, Lung, and Blood Institute at the National Institutes of Health established the Pediatric Heart Network (PHN) in 2001 to stimulate multi-center collaboration and clinical studies in children and adults with congenital heart disease (CHD) and pediatric acquired heart disease.

**Methods:**

The PHN developed a flexible infrastructure for multi-center collaborative clinical research in children and adults with CHD and pediatric acquired heart disease. The objectives of the PHN are to improve health outcomes in individuals of all ages with CHD and pediatric acquired heart disease, to disseminate findings to improve treatment options and standards of care, to train and educate new investigators, and to support families during the conduct of clinical research.

**Results:**

To date, the PHN has conducted 30 studies, including 13 clinical trials, across over 60 sites and has enrolled over 10,000 participants. PHN studies have impacted clinical practice and guidelines in CHD and have supported the career development of young investigators, research nurses, and study coordinators. None of this would have been possible without the many partnerships with patient advocacy organizations, the U.S. Food and Drug Administration, a variety of industry collaborators and clinical registries. PHN studies have leveraged registry data to improve efficiency, minimize burden and reduce errors in data collection.

**Conclusion:**

The PHN’s success is due to fostering collaboration across pediatric cardiology centers, creating a clinical research infrastructure that can adapt to different types of studies, and emphasizing career development of young investigators and research coordinators. This paper will summarize the PHN’s history, partnerships, use of clinical registries, future directions, and ways to get involved.

## 1 History and Development

Congenital heart disease (CHD) survival significantly improved in the decades leading up to the establishment of the Pediatric Heart Network (PHN). This improvement was due to multiple factors, including improvements in imaging, the establishment of critical care units, and innovations in surgical and catheter-based interventions. There were many innovative therapeutic approaches devised by individuals or small groups of surgeons and cardiologists. However, in the 1990s, there were still few multicenter research collaborations and pediatric cardiovascular clinical trials. This lack hindered the development of evidence-based clinical guidelines, long available in adult cardiology [1]. Given the rarity of congenital heart diseases, it is often difficult to answer important questions facing the field at just one center. Therefore, the National Heart, Lung, and Blood Institute established the PHN in 2001 to create an infrastructure to fill this gap by supporting multi-center clinical studies and trials in pediatric cardiovascular disease.

The objectives of the PHN are the following:

1. To improve health outcomes in individuals of all ages with congenital heart disease or pediatric acquired heart disease
2. To disseminate collaborative findings as the basis for improved evidence-based treatment options and standards of care
3. To train and educate new investigators
4. To provide support for families during the conduct of excellent, ethical clinical research

In its earliest iteration, the PHN launched two clinical trials, one studying intravenous steroids in addition to standard treatment for Kawasaki Disease (KD) and the other evaluating whether enalapril improved growth and heart function in infants with single ventricle heart disease (ISV) [2,3]. The PHN subsequently brought together pediatric cardiologists and cardiac surgeons from across the United States and Toronto, Canada to collaborate on the PHN’s first surgical clinical trial. At that time, one of the major questions facing the field was around the best palliation strategy for Hypoplastic Left Heart Syndrome (HLHS). Surgeons had started to perform the right ventricle to pulmonary artery shunt (RVPA/Sano shunt) in lieu of the modified Blalock-Taussig-Thomas shunt (mBTTs). However, initial studies on this modification yielded mixed results. The PHN launched the Single Ventricle Reconstruction (SVR) trial in May 2005 to compare these two surgical strategies. This landmark trial, which was the PHN’s first surgical clinical trial, successfully enrolled 555 infants with HLHS [4]. The trial results showed that while transplantation-free survival at 12 months was higher with the RVPA shunt than with the mBTTs, longer term follow-up consistently demonstrated no significant difference in transplant-free survival between the two groups [4]. Arguably as important as the immediate results of the study, the SVR trial demonstrated the PHN’s unique ability to foster collaboration between pediatric cardiologists and cardiac surgeons from across the country to conduct important, challenging multi-center trials. Prior to the SVR trial, many were skeptical that pediatric cardiologists and cardiac surgeons would be willing to randomize their patients between surgical shunt types, despite the equipoise in the field. The early success of the PHN model set a benchmark for subsequent studies. Many participants in the SVR trial continue to be followed 20 years later, and have helped inform understanding of single ventricle long-term outcomes including survival, transplant, cardiac function, and neurodevelopment [5–7].

Beyond their immediate results, the KD, ISV and SVR trials demonstrated that multi-center collaboration could result in more efficient enrollment, shorter trial times, more representative participation, and broader clinical impact. The established PHN infrastructure also allowed for important long-term follow up on SVR participants, which is a challenge with other grant funding timelines, in which trial infrastructure is set up only for the duration of that particular grant.

In the early planning phase, the PHN recognized the challenges of conducting randomized clinical trials in children with CHD due to the lack of clinically significant, frequently occurring, primary outcome variables. Despite an interest in using surrogate endpoints in trials, there is often not sufficient evidence to link the changes in surrogate outcomes to clinical outcomes [8]. The PHN’s Fontan Cross-Sectional Study was designed to identify the association between reported health status and laboratory measures of ventricular dimensions and performance in children with Fontan circulation [8]. This observational study informed the planning and provided estimates of event rates for the outcome measures selected for the Fontan Udenafil Exercise Longitudinal (FUEL) trial, a randomized, double-blind, placebo-controlled phase III trial that evaluated whether udenafil improved exercise capacity in adolescents who had undergone Fontan operation [9]. In the future, CHD registries may serve as an important resource for identifying appropriate clinical trial endpoints and understanding event rates in order to appropriately power clinical trials.

## 2 Target Population and Scope (Research, Quality Improvement)

The PHN provides a flexible infrastructure for multi-center collaborative clinical research in children and adults with CHD and pediatric acquired heart disease. The PHN supports clinical studies evaluating medical, interventional and surgical therapies, and attempts to identify health disparities in CHD outcomes. The PHN also provides clinical research experience for research nurses, study coordinators, fellows and junior faculty.

The PHN supports phase I, II and III clinical trials, observational studies, and multiple related activities including biorepository cores, data science initiatives and a social determinants of health committee. The unique involvement of study coordinators and nurses in protocol development and feedback has contributed to the PHN’s long-term sustainability and success. Study coordinators and nurses provide a unique, important perspective on every aspect of developing a study protocol, including considerations around engaging participants, consenting and adverse event reporting. The PHN has prioritized incorporating study coordinators into all protocol development working groups and the majority of PHN committees. In addition, the Nursing Research Committee has successfully led six ancillary studies and one clinical trial. The PHN is one of the only NIH networks supporting nursing-led research and providing clinical research leadership and mentorship opportunities for nurses and study coordinators. As just one example, the PHN supported the first nursing-led pilot study and subsequent phase III randomized trial of a passive range of motion exercise program for infants with hypoplastic left heart syndrome and other single right ventricle anomalies [10].

## 3 Governance and Partnerships

**Figure 1:**
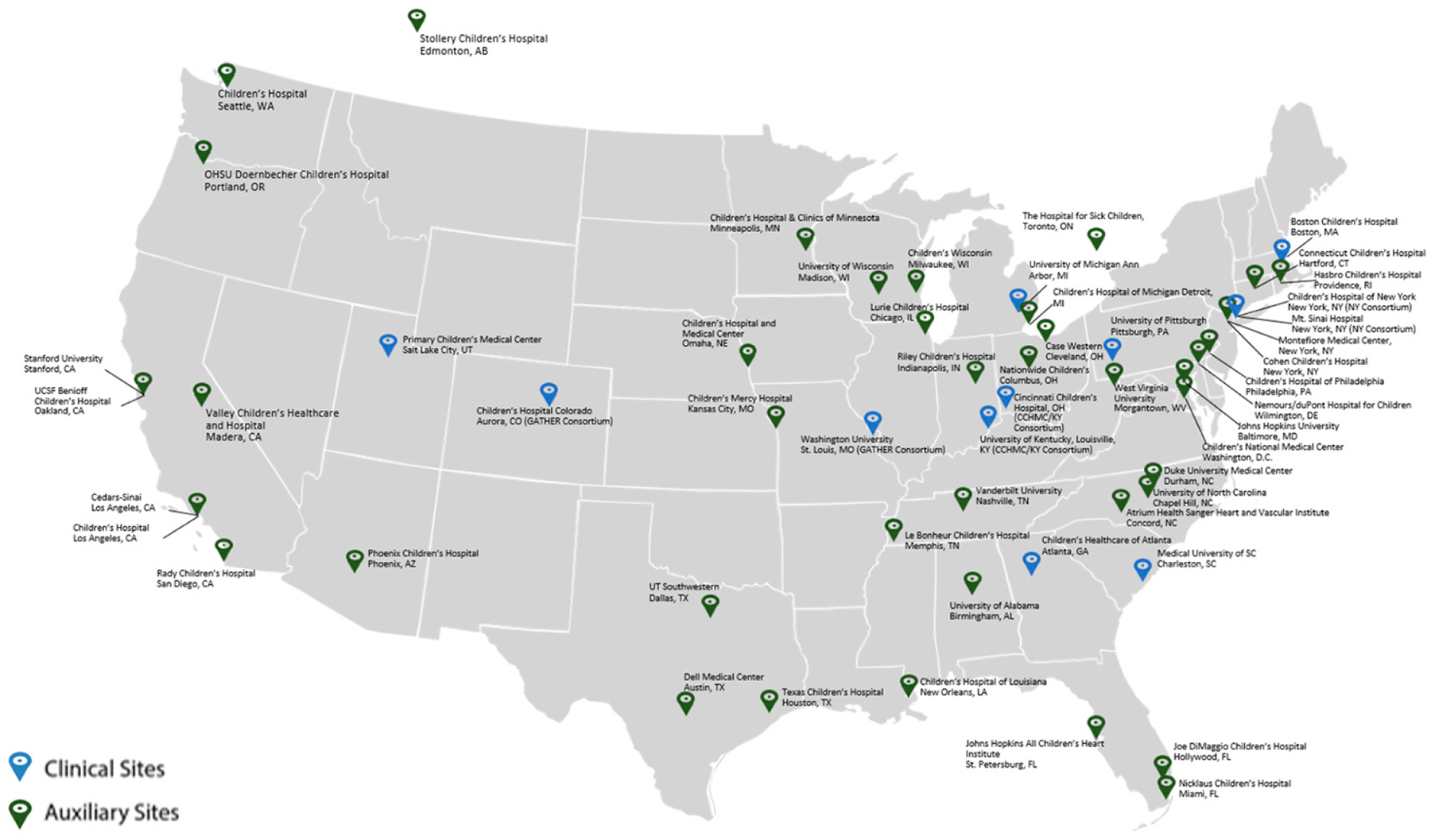
Pediatric heart network sites map (as of publication date).

The PHN is a cooperative endeavor composed of a data coordinating center (DCC), nine core clinical research centers (CRCs), of which three are two-site consortia, auxiliary sites employed to obtain sufficient numbers of participants for a given study, and NHLBI (Fig. 1). Investigators from the sites work collaboratively to propose, develop, and refine the protocols, as well as implement the clinical trials or studies. The DCC provides overall Network coordination by supporting study activities, quality assurance, and protocol development, and providing statistical guidance and data analysis. The PHN Executive Committee is the main governing body of the PHN and is composed of one Principal Investigator from each of the core CRCs, the DCC staff, chairs of select PHN Committees, the Study Coordinator chair and the NHLBI program team. The PHN Steering Committee is composed of PHN investigators from both the CRCs and auxiliary sites, study coordinators, the DCC staff, and NHLBI program staff.

**Figure 2:**
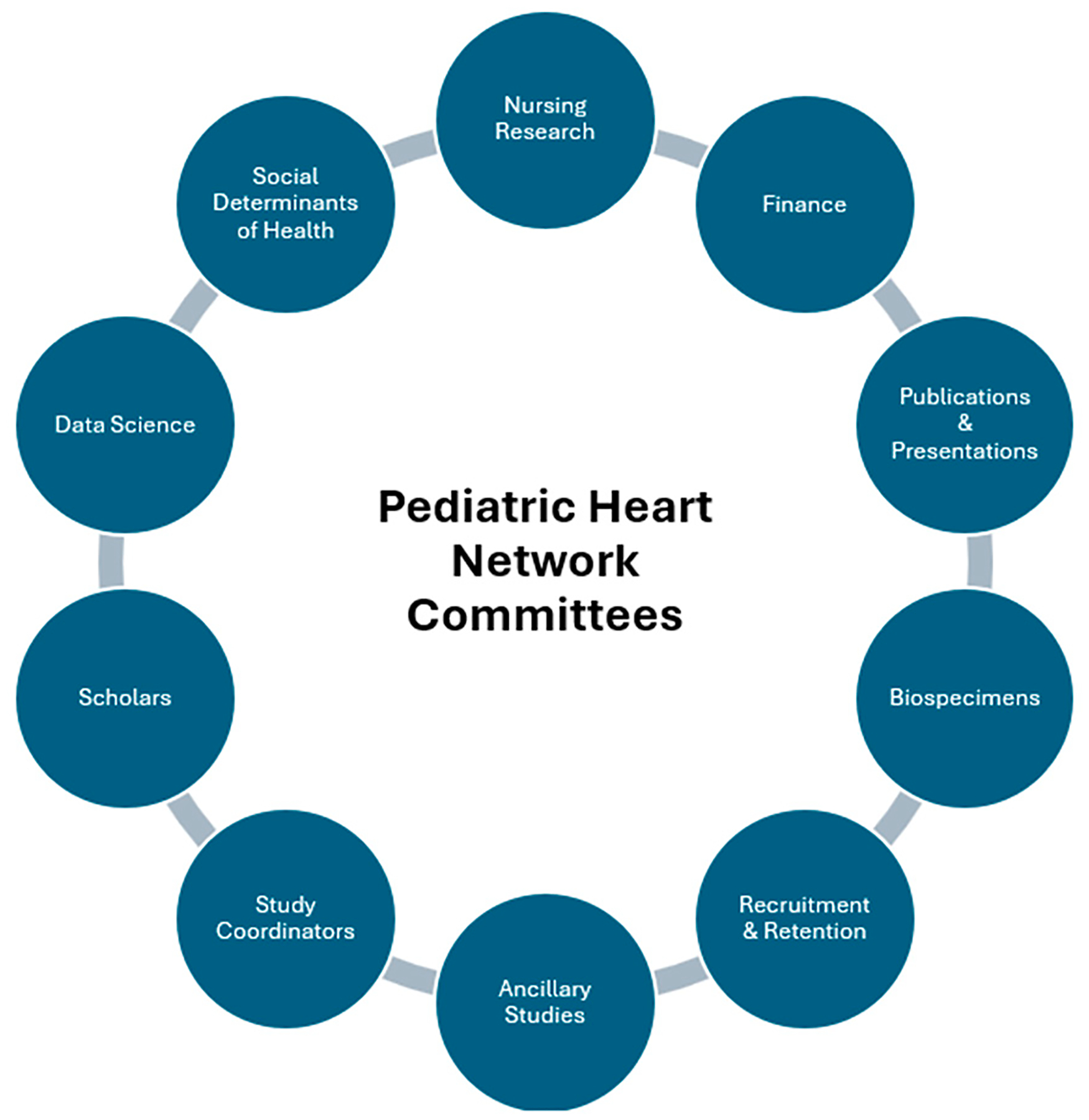
Current pediatric heart network committees.

The PHN’s administrative and scientific work is divided among several committees whose membership is drawn from the CRCs, auxiliary sites, the DCC and NHLBI (Fig. 2). The PHN has a NHLBI-appointed Protocol Review Committee tasked with providing independent peer review, including critical evaluation, feedback, and approval of proposed protocols. The NHLBI-appointed Data and Safety Monitoring Board (DSMB) monitors study participant safety and reviews performance of each study.

**Figure 3:**
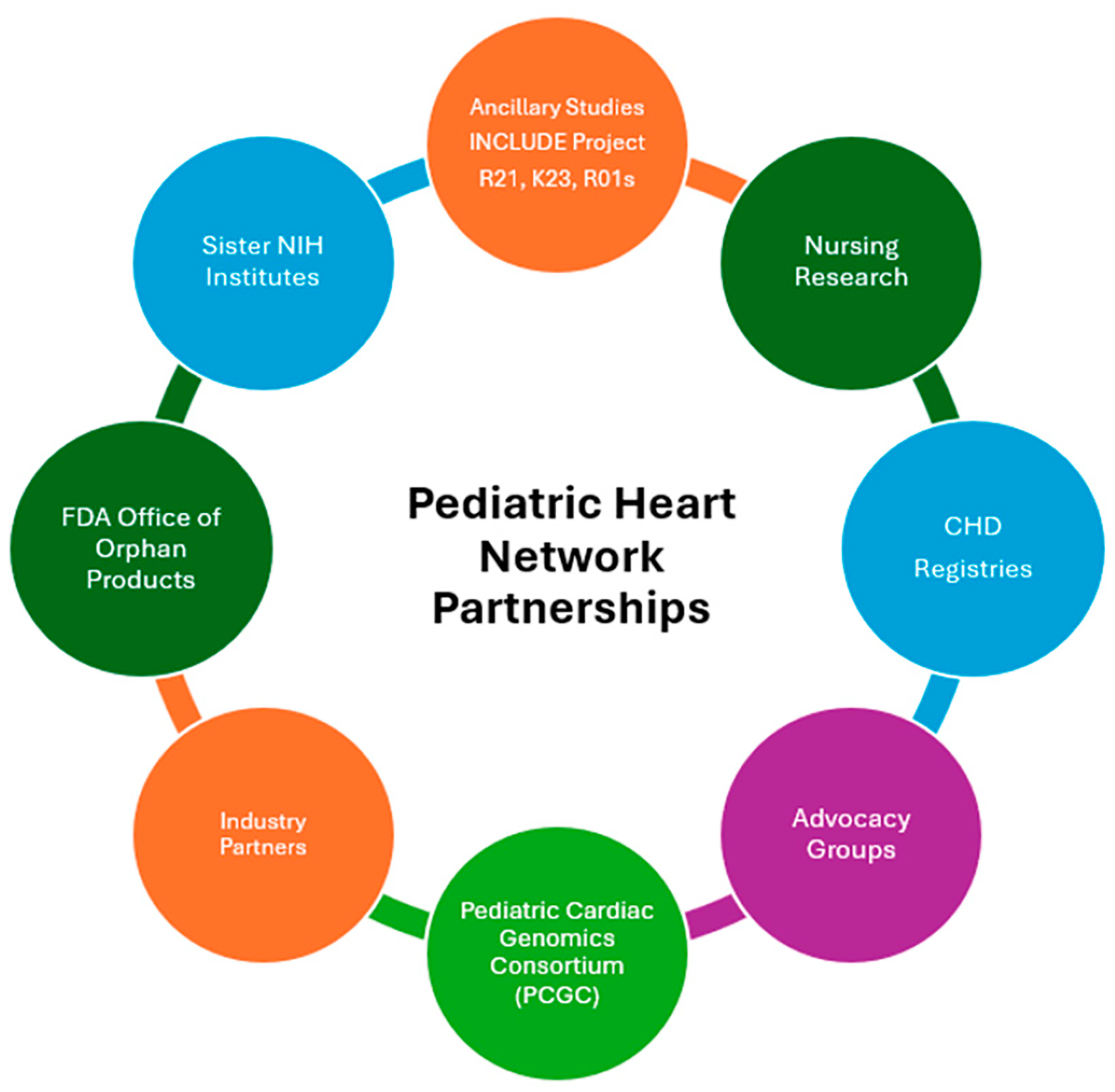
Pediatric heart network collaborations.

The PHN’s work has benefitted from numerous collaborations, including with patient advocacy organizations, the U.S. Food and Drug Administration, other NIH-funded consortia and projects, a variety of industry collaborators, and clinical registries (Fig. 3). Patient advocacy organizations have provided valuable feedback on clinical trial protocols, assisted the PHN in identifying scientific priorities for study, and helped with publicizing opportunities for participation in PHN research. These organizations also help disseminate important results of PHN studies to the CHD community.

The NHLBI-funded Pediatric Cardiac Genomics Consortium (PCGC) has worked with the PHN Biospecimens Committee to provide genetic expertise, as well as sequencing and analysis of biospecimens from PHN studies. The NIH INvestigation of Co-occurring conditions across the Lifespan to Understand Down syndromE (INCLUDE) Project (nih.gov/include-project) [11] funded a PHN study to better understand the neurodevelopmental and behavioral impact of CHD repaired in infancy on outcomes in school-aged children with Down syndrome. This study leveraged a portion of the cohort who had enrolled previously in a PHN study called the Residual Lesion Score Study [12].

The PHN infrastructure has also proven to be attractive to industry partners interested in the pediatric cardiology space, leading to several successful collaborations. In a collaboration with Bristol Myers Squibb (BMS), the PHN completed a phase 2, open-label trial of apixaban in children with heart disease requiring thromboprophylaxis [13]. The trial found that bleeding and thromboembolism were infrequent in both the apixaban and standard of care group, supporting the use of apixaban as an oral alternative that does not require monitoring to standard of care (vitamin K antagonists and low-molecular-weight heparin) [13].

The PHN has also successfully collaborated with several clinical registries, including the Pediatric Cardiac Critical Care Consortium (PC4) and the Congenital Cardiac Research Collaborative (CCRC). Additional details on these partnerships are discussed below.

## 4 Present State (Feedback to Participants, Audits and Benchmarking)

The PHN has conducted 30 clinical studies across over 60 sites, both in the United States and internationally, and has enrolled over 10,000 participants in its various studies and trials. Thirteen of these studies were clinical trials. The PHN is in the process of developing and launching three new clinical trials, including two with behavioral interventions and one drug trial for patients with biventricular heart failure. The PHN continues to focus on career development and building on existing collaborations, while also exploring opportunities in data science and health disparities research. The PHN recently established the Data Science Committee to explore opportunities for optimizing data extraction, integration, and standardization across multiple sources, including registries, for clinical studies. The recently created Social Determinants of Health (SDoH) Committee is tasked with developing a set of standard SDoH measures to be collected across studies, as well as recommending specific measures based on individual study considerations (e.g., participant age).

While the PHN has continually sought input from the CHD community, a Recruitment and Retention Committee was recently created in order to seek additional, more formal feedback throughout protocol development and recruitment. In addition, a CHD community member (parent of a child with CHD or an adult with CHD) participates as a member of the study team for two of the clinical trials that are in the planning phase.

The PHN maintains a strong emphasis on career development for early-career physician scientists interested in congenital cardiology or acquired pediatric heart disease research. The PHN has supported 37 scholars since 2013, with three scholars also supported by the American Heart Association for projects focused on adult congenital heart disease and six scholars supported by the NIH INCLUDE Project for projects focused on Down syndrome. Once a year, the PHN hosts a “Career Day” event for early-career investigators from existing PHN sites to learn about the PHN and how to pursue a physician-scientist career. Career Day includes several interactive sessions, including an overview of NIH grants and grant mechanisms and, opportunities to get involved in PHN research. Career Day participants develop networking and mentorship relationships with established PHN investigators, and receive career and research advice from a panel of leaders in pediatric cardiology clinical research. In addition, the PHN also fosters career development of network study coordinators and research nurses through a “Core Day.” Core Day allows for study coordinators to share experiences, learn best practices and explore career opportunities in research. The attendees from both Career Day and Core Day are also invited to stay for the subsequent two days of the PHN Steering Committee to learn more about ongoing clinical studies and other PHN activities.

In regard to audits and benchmarking, PHN study enrollment and overall performance is tracked both at the DCC, as well as by NHLBI with oversight by the PHN DSMB. The PHN has provided feedback to the CHD community on study results and opportunities to participate in PHN research through various approaches, including newsletters, webinars, the PHN website and dissemination by advocacy organizations.

## 5 Examples of Where Registry Data Informed PHN Clinical Studies

Over the last 20 years, there has been a growth in the number of pediatric cardiology and congenital heart disease registries, and number of participating centers. In the planning stages of a clinical trial, registries can provide valuable information on number of potentially eligible participants and event rates. This information has aided the PHN in informing power calculations and deciding on number of participating sites. Registry data also provides the opportunity to collect a wealth of data on study participants without adding significant research coordinator burden. In addition, registries typically follow patients over much longer periods than is feasible in a clinical trial. This may allow registries to capture long-term benefits or harm that may have not been captured in short-term trial follow up. The PHN has two studies that have integrated registries directly into their data collection processes.

The PHN Residual Lesion Score (RLS) Study (2015–2017) was the first PHN study to pilot the use of registry data [12]. RLS was a prospective, observational cohort study to understand the association between residual lesions following congenital heart surgeries and both early and mid-term clinical outcomes [14,15]. Data collection came from two sources: (1) the traditional method of manual collection by trained research staff and (2) from the extraction of existing data already collected by sites for submission to the Society of Thoracic Surgeons-Congenital Heart Surgery Database [15]. The reliability of the local registry data was verified through a retrospective audit of 500 participants, and 94.7% of the data elements were found to be complete and accurate [14]. RLS aimed to collect approximately 240 individual variables (approximately 10% of the total study variables) from site’s local registry data. The team created site-specific queries used to extract registry data from consented participants on a monthly basis for about 2 years. Study coordinators at each site would review data query results, remove protected health information (PHI), and share with the PHN DCC. Study coordinators also collected other non-registry variables through traditional chart review. Additional quality assurance processes were also in place at the DCC to ensure data integrity [15].

A survey evaluating perceptions of utilizing registry data in RLS demonstrated that the majority (57%, *n* = 31/54) of respondents agreed that using local registry data saved the research staff time. The survey also indicated that the majority of respondents (71%, *n* = 37/54) thought using local registry data instead of standard data collection methods would save time in future studies [15]. The main challenges identified by survey respondents were that some sites did not routinely collect all of the data fields, programming of local data abstraction can be complicated and time-consuming, registry data may be collected and submitted less frequently than what is required for a study or trial, and that PHI had to be manually removed by study coordinators [15].

Another PHN study to leverage registry data was the Comparison of Methods of Pulmonary blood flow Augmentation in neonates: Shunt versus Stent (COMPASS) Trial. COMPASS was a multicenter, randomized interventional trial comparing a ductal artery stent versus a systemic-to-pulmonary artery shunt in neonates with ductal-dependent pulmonary blood flow. This trial’s data management plan used two Cardiac Network United-affiliated registries, the PC4 and the CCRC, to collect most of the data elements for the study, with the exception of time-sensitive data like consent and adverse events [16]. This method minimized data collection burden by decreasing the number of case report forms that study coordinators had to complete. Each registry has a data auditing process to ensure accurate data capture. In addition, the CCRC serves as a “Companion Registry” to track patients who were eligible for the trial but weren’t enrolled for a variety of reasons [16].

Leveraging registries in this way can serve as a model for reducing errors and improving efficiency of data collection for clinical studies. COMPASS was able to extract data directly from PC4 and CCRC, whereas RLS collected registry data from individual sites. Several themes emerged from the RLS [15] and the COMPASS experiences in using registries for clinical studies:

1. Registry and research partners should be involved from the start for successful integration of registry data into clinical study processes.
2. Registries and clinical studies may have different data variables of interest and timelines, both of which are important to consider in the design phase.
3. Creating simplified programming and processes for extracting registry data should be considered.
4. Extracting data directly from a registry is a strategy to reduce study coordinator burden and avoid issues with inadvertent sharing of PHI.

The PHN’s experience demonstrates that while initial registry integration in clinical studies presents logistical and planning challenges, the benefits of efficiency, data richness, long-term follow-up data, and reduced coordinator burden are substantial. The PHN will continue to pursue collaborations with CHD registries in the future.

## 6 Future State: Opportunities, Challenges, Data Gaps

In 2021, NHLBI convened a virtual workshop to identify current gaps and future research opportunities to reduce morbidity and mortality related to CHD across the lifespan and to inform the future of the PHN [17]. Several of the challenges identified are focus areas for the PHN today.

Insufficient collaboration across data platforms limits the ability to track patients longitudinally and identify evolving outcomes [17]. For example, a baby with CHD enrolled in a PHN clinical trial will have immediate outcome data collected; however, it is challenging and costly to collect long-term outcomes even 5 to 10 years later. The SVR Trial has served as a model for collecting long-term outcome data, but it has required a significant financial investment by the PHN as well as study coordinator and investigator time to do so. In the future, CHD registries could help in providing longitudinal data on participants in clinical trials. This would require finding a way to link participants in clinical trials to their records in CHD registries. In addition, it will be important to find a way to link participants across registries in order to understand their full picture. For example, in a patient with hypoplastic left heart syndrome who participates in a clinical trial as an infant and eventually receives a heart transplant many years after a Fontan palliation, it would be important to have surgical, inpatient, outpatient, neurodevelopment and heart transplant registry data to understand the full picture. The PHN is actively exploring data tokenization and linkage protocols to connect trial participants with existing registries.

The workshop also noted a lack of comprehensive clinical research strategies, particularly regarding research consideration outside the scope of clinical pediatric cardiology expertise, including impact of genetics and psychosocial factors that are important to patients and families. Inefficient research designs and lack of transformational research were also noted as challenges in this workshop [17]. This challenge has been met by the PHN in developing two clinical trials during the current funding cycle of the PHN. Both trials are led in part by psychologists with expertise in congenital heart disease, and both studies are evaluating mental health and quality of life considerations in CHD. One of the trials employs a pragmatic approach through a telemedicine mental health intervention to study resilience in adolescents with moderately or severely complex CHD. The second trial has an innovative platform design to study a mental health and breastfeeding intervention in mothers and their infants with critical CHD. Expanding the PHN to integrate psychologists has led to innovation in the research questions asked and tools used. To incorporate psychosocial factors more effectively, the SDoH Committee is creating standardized measures to be incorporated across all future PHN trials.

### Limitations

We acknowledge some limitations of the PHN, such as its limited geographic footprint, funding dependency, and difficulty engaging representative populations in research. The PHN has involved primarily North American sites given logistical considerations and its funding structure supported by United States taxpayer dollars. Moreover, maintaining the PHN depends on ongoing funding from the National Heart, Lung, and Blood Institute, National Institutes of Health, which has many important competing scientific demands on its budget. As in many other research studies, the PHN has struggled to enroll and retain participants who are representative of the CHD and pediatric cardiology patient population. To that end, the PHN has created the Recruitment and Retention Committee to inform protocol development and participant and community engagement. To address these limitations, it is imperative that PHN collaborate with other key players in our field, such as registries.

## 7 How to Get Involved

The PHN prioritizes engaging investigators at all levels interested in CHD research. One way to get involved is to join the team at a PHN core or auxiliary site. There are often opportunities for helping with currently enrolling studies, joining writing committees, or proposing writing topics.

The PHN also publishes public use data sets on all of its studies (PediatricHeartNetwork.org), which are available for any interested investigator to analyze (Table 1). There is also a catalog of publicly available biospecimens on the website that could be used for future-omics analyses.

The PHN encourages investigators to propose new multi-center clinical studies and trials that would advance the field of pediatric and congenital cardiology, adult congenital cardiology, or congenital cardiac surgery. There is also the opportunity to propose ancillary studies for investigations that are not part of a main PHN protocol but obtain additional data on PHN participants to answer important scientific questions. Ancillary studies are funded outside the PHN grant funds, typically by funding from investigator-initiated NIH research grants, foundations, institutions, or other private sources. Both new PHN studies and ancillary studies can be proposed by any investigator by collaborating with a PHN core site investigator in the development of their proposal. The PHN also continues to look for opportunities for collaboration with existing clinical registries for PHN studies in the planning phases as well as the development of new proposals.

## Figures and Tables

**Table 1:** Pediatric heart network public use datasets.

Study Dataset	Brief Description
Fontan Cross-sectional Study	Observational study evaluated health status, exercise capacity, laboratory results and cardiac imaging in children with Fontan circulation.
Collaborative Learning Study	Quality improvement study of a clinical practice guideline aimed at increasing rates of early extubation in infants with tetralogy of Fallot and aortic coarctation.
Infant Single Ventricle Trial	Randomized trial evaluating enalapril versus placebo in infants with single ventricle heart disease.
Trial of Pulse Steroid Therapy in Kawasaki Disease	Randomized trial evaluating whether adding steroids to standard treatment in Kawasaki disease would improve coronary artery outcomes.
Marfan Trial	Randomized trial comparing atenolol to losartan on aortic root growth over three years.
Single Ventricle Reconstruction Trial (SVR & SVR II)	Randomized trial comparing right ventricular to pulmonary artery shunt to modified Blalock-Taussig-Thomas shunt in infants with single, morphologically right ventricle undergoing Norwood procedure. SVR II is a longitudinal follow-up observational study up to 6 years of age.
Variability of Echocardiogram Left Ventricular Mass, Volume and Ejection Fraction in Pediatric Patients with Dilated Cardiomyopathy	Observational study of clinical data and echocardiographic indices in individuals with known or suspected dilated cardiomyopathy aged 0–22 years.
Echocardiogram Z-score and Normal Electrocardiogram Study	Observational study of echocardiography measures and ECGs from children without heart disease with the objective of establishing normal values (Z-scores) for common echocardiogram measurements.
DO IT!	Randomized trial comparing Pitavastatin to placebo on vascular measures in adolescents with excess adiposity and combined dyslipidemia of obesity.

## Data Availability

There was no new data presented in this manuscript. PHN data is made publicly available after publication and as Public Use Datasets on the PHN website.
